# Draft Genome Sequence of the Drought-Tolerant Plant Growth-Promoting Rhizobacterium Bacillus altitudinis UKM RB11, Isolated from Upland Paddy Rhizosphere

**DOI:** 10.1128/mra.00617-22

**Published:** 2022-09-15

**Authors:** Emmyrafedziawati Aida Kamal Rafedzi, Mohd Faizal Abu Bakar, Izwan Bharudin, Shazilah Kamarudin, Farah Diba Abu Bakar, Abdul Munir Abdul Murad

**Affiliations:** a Department of Biological Sciences and Biotechnology, Faculty of Science and Technology, Universiti Kebangsaan Malaysia, Bangi, Selangor, Malaysia; b Soil Sciences, Water and Fertilizer Research Centre, Malaysian Agricultural Research and Development Institute, Serdang, Selangor, Malaysia; c Malaysia Genome and Vaccine Institute, National Institute of Biotechnology Malaysia, Kajang, Selangor, Malaysia; University of Arizona

## Abstract

Asia’s paddy areas endure tropical and equatorial monsoon climates and are prone to drought stress. The drought-tolerant plant growth-promoting rhizobacterium (PGPR) strain Bacillus altitudinis UKM RB11 was isolated from upland paddy soil in Malaysia. Its 3.7-Mb genome sequence contains numerous genes involved with tolerance to drought and high temperatures and plant growth promotion.

## ANNOUNCEMENT

Using plant growth-promoting rhizobacteria (PGPR) can improve the development, stress tolerance, and general performance of water-stressed fields and greenhouse crops. PGPR stimulate plant development directly through biofertilization, plant hormone production, and rhizoremediation (1). Additionally, PGPR indirectly reduce plant diseases by preventing the growth of harmful bacteria and fungi (2).

Bacillus altitudinis UKM RB11 was isolated from upland paddy rhizosphere soil in Bau, Sarawak, Malaysia (1°29′11.6″N, 110°00′55.3″E). Soil (10 g) and cleaned rice root were added to 90 mL sterile distilled water. The samples were then shaken for a few minutes to homogenize them. Each sample underwent serial dilution of 10^−7^, and 50 μL of each dilution was pipetted onto a nutrient agar (NA) plate. A single colony was selected and streaked onto NA plates to grow at 50°C for 24 h. This strain (named UKM RB11) showed the ability to grow at high temperatures, utilize 1-aminocyclopropane-1-carboxylate (ACC) substrate as an energy source, and produce the enzyme ACC deaminase, which can degrade ACC to reduce plant ethylene levels under stress (3). ACC deaminase activity was detected in a 24-h culture with 1.04 mmol α-ketobutyrate h^−1 ^mg^−1^ protein (4). Strain UKM RB11 produces plant phytohormone and indole acetic acid (IAA) (5), solubilizes phosphate (6), and fixes nitrogen (7). UKM RB11 and two other strains developed a mixed PGPR and promoted rice (Oryza sativa) growth in an experiment conducted in a glasshouse under drought conditions.

Single colonies of Bacillus altitudinis UKM RB11 pure cultures were extracted to obtain genomic DNA using the GenElute bacterial genomic DNA kit (Sigma-Aldrich, UK), starting from a fresh overnight culture in Luria Bertani (LB) broth incubated at 30°C with shaking at 160 rpm. Using the NanoDrop spectrophotometer, the DNA concentration was measured at 36.5 μg/mL (*A*_260/280_ = 2.1). Subsequently, the genomic DNA was sequenced using the Illumina NovaSeq platform and the NEBNext Ultra II DNA library prep kit. A total of 3,980,425 paired-end reads were generated, with a total size of 1.2 Gb.

Sequence assembly was conducted using the Velvet v1.2.10 assembler (8), resulting in an assembly with 144 contigs. The contigs were combined into scaffolds using SSPACE Standard v3.0 (9), RagTag v1.1.0 (10), and MeDuSa v1.6 (11), using Bacillus altitudinis strain SGAir0031 (GenBank accession number NZ_CP022319.2) as the reference genome. The final version of the draft genome assembly resulted in a single scaffold with 91 contigs. The total genome size is 3,767,351 bp, with an average GC content of 41.03%. The UKM RB11 draft genome is represented as a circular chromosome but is still incomplete, and the circularization was not confirmed. The genome was subsequently annotated using the NCBI Prokaryotic Genome Annotation Pipeline (PGAP) v6.1 (12). A total of 3,911 genes were identified, including 3,823 protein-coding sequences (CDSs), of which 3,783 (97%) had predicted functions, and 40 non-protein-coding genes. The average nucleotide identity (ANI) between *B. altitudinis* UKM RB11 and *B. altitudinis* strain SGAir0031 was 98.4%, calculated using the EzBioCloud ANI calculator (13). Default parameters were used for all software unless otherwise specified.

The genome of *B. altitudinis* UKM RB11 includes various genes that have putative functions related to stress and heat tolerance. The UKM RB11 genome contains multiple genes involved in plant growth promotion, including IAA biosynthesis via a tryptophan-dependent pathway, nitrogen metabolism, phosphate solubilization, siderophore biosynthesis, and hydrogen sulfide synthesis. Additionally, a gene for motility, chemotaxis, and rhizosphere colonization was identified in the UKM RB11 genome. Several genes encoding antioxidant enzymes and volatile organic compounds (VOCs), genes encoding proteins that can inhibit the growth of other microorganisms, and several genes that regulate spore formation were also found in the UKM RB11 genome.

### Data availability.

The draft whole-genome sequence of *B. altitudinis* UKM RB11 is available through NCBI under BioProject accession number PRJNA821328, SRA accession number SRX16400562, and GenBank accession number CP094654.
